# STRESSORS OF FILIAL ANXIETY REGARDING ANTICIPATED CARE OF OLDER PARENTS: EVIDENCE FROM CHINESE ADULT CHILDREN

**DOI:** 10.1093/geroni/igad104.2227

**Published:** 2023-12-21

**Authors:** Chang Liu, Xue Bai

**Affiliations:** The Hong Kong Polytechnic University, Hong Kong, Hong Kong; The Hong Kong Polytechnic University, Hong Kong, Hong Kong

## Abstract

Evidence has shown that the anticipation of providing care for a parent in the future can be anxiety-provoking for adult children, especially for one-child generations in China. Filial anxiety may influence adult children’s transition to the caregiver role and may bring about heightened caregiving stress. However, this phenomenon remains insufficiently studied among Chinese adult children. Drawing on the Stress Process Model and proactive coping theory, this study examined how primary stressors (i.e., parent’s declining health, adverse psychological health, and lack of eldercare resources), anticipatory stressor (i.e., anticipated parental care needs), and psychosocial resources (i.e., sibling number, the value of filial obligation, intergenerational relationship, work stress, and family stress) influenced the multiple domains of filial anxiety (i.e., Filial Anxiety-Ability, Filial Anxiety-Responsibility, and Filial Anxiety-Welfare). A face-to-face questionnaire survey was conducted with 530 Chinese adult children aged between 26 and 40 years in Shenzhen, China. Descriptive analyses and regression analyses were conducted. Results demonstrated that Chinese adult children experienced a higher level of overall filial anxiety than their Western counterparts and reported a particularly high level of filial anxiety about their parent’s welfare. Adult children with higher work and family stress, with fewer siblings, and whose parents lack eldercare resources were more likely to experience a high level of filial anxiety. This study systematically investigated the multiple stressors of filial anxiety. The findings have implications for the development of effective interventions to reduce adult children’s future caregiving concerns and better prepare them for their older parents’ future care needs.

